# Multi-ancestry fine mapping implicates *OAS1* splicing in risk of severe COVID-19

**DOI:** 10.1038/s41588-021-00996-8

**Published:** 2022-01-13

**Authors:** Jennifer E. Huffman, Guillaume Butler-Laporte, Atlas Khan, Erola Pairo-Castineira, Theodore G. Drivas, Gina M. Peloso, Tomoko Nakanishi, COVID-19 Host Genetics Initiative, Andrea Ganna, Anurag Verma, J. Kenneth Baillie, Krzysztof Kiryluk, J. Brent Richards, Hugo Zeberg

**Affiliations:** 1 Massachusetts Veterans Epidemiology Research and Information Center, VA Boston Healthcare System, Boston, MA USA; 2 Departments of Medicine, Human Genetics, Epidemiology, Biostatistics and Occupational Health, McGill University, Lady Davis Institute, Jewish General Hospital, Montréal, Québec Canada; 3 Division of Nephrology, Department of Medicine, Vagelos College of Physicians & Surgeons, Columbia University, New York, NY USA; 4 Roslin Institute, University of Edinburgh, Edinburgh, UK; 5 Medical Research Council Human Genetics Unit, Institute of Genetics and Cancer, University of Edinburgh, Western General Hospital, Edinburgh, UK; 6 Department of Genetics, Perelman School of Medicine, University of Pennsylvania,, Philadelphia, PA USA; 7 Division of Human Genetics, Department of Pediatrics, Children’s Hospital of Philadelphia, Philadelphia, PA USA; 8 Division of Translational Medicine and Human Genetics, Department of Medicine, Perelman School of Medicine, University of Pennsylvania, Philadelphia, PA USA; 9 Department of Biostatistics, Boston University School of Public Health, Boston, MA USA; 10 Department of Human Genetics, McGill University, Montréal, Québec Canada; 11 Lady Davis Institute, Jewish General Hospital, McGill University, Montréal, Québec Canada; 12 Kyoto-McGill International Collaborative School in Genomic Medicine, Graduate School of Medicine, Kyoto University, Kyoto, Japan; 13 Japan Society for the Promotion of Science, Tokyo, Japan; 14 Institute for Molecular Medicine Finland, Helsinki Institute of Life Science, University of Helsinki, Helsinki, Finland; 15 Massachusetts General Hospital, Harvard Medical School, Boston, MA USA; 16 Corporal Michael Crescenz VA Medical Center, Philadelphia, PA USA; 17 Institute for Genomic Medicine, Columbia University, New York, NY USA; 18 Department of Twin Research, King’s College London, London, UK; 19 Max Planck Institute for Evolutionary Anthropology, Leipzig, Germany; 20 Department of Neuroscience, Karolinska Institutet, Stockholm, Sweden

**Keywords:** Infectious diseases, Genetic association study, Immunogenetics

## Abstract

The *OAS1/2/3* cluster has been identified as a risk locus for severe COVID-19 among individuals of European ancestry, with a protective haplotype of approximately 75 kilobases (kb) derived from Neanderthals in the chromosomal region 12q24.13. This haplotype contains a splice variant of *OAS1*, which occurs in people of African ancestry independently of gene flow from Neanderthals. Using trans-ancestry fine-mapping approaches in 20,779 hospitalized cases, we demonstrate that this splice variant is likely to be the SNP responsible for the association at this locus, thus strongly implicating *OAS1* as an effector gene influencing COVID-19 severity.

## Abstract (web summary)

Multi-ancestry fine-mapping of the *OAS1/2/3* region shows that a splice site variant in *OAS1* is likely responsible for the association of this locus with the risk of severe COVID-19.

## Main

The COVID-19 pandemic has impacted the world for over a year. During this period, several large international efforts^1–5^ have been launched to identify the genetic determinants of COVID-19 susceptibility and severity. These efforts have identified more than a dozen genomic regions associated with severe COVID-19. However, the causal variants in these regions are yet to be identified, hampering our ability to understand COVID-19 pathophysiology.

When risk haplotypes are long, it is more challenging to disentangle causal genetic variants due to linkage disequilibrium (LD). This is especially problematic for haplotypes derived from Neanderthals and Denisovans, which often span several tens of kb or more. Two notable COVID-19 examples are the major risk locus on chromosome 3 (3p21.31) and the *OAS1/2/3* locus on chromosome 12 (12q24.13), both carrying haplotypes of Neanderthal origin^6,7^. The *OAS* genes encode enzymes catalyzing the synthesis of short polyadenylates, which activate ribonuclease L that in turn degrades intracellular double-stranded RNA and triggers several other antiviral mechanisms^8^. The protective Neanderthal-derived haplotype confers approximately 23% reduced risk of becoming critically ill on infection with SARS-CoV-2 (ref. ^3^). Supporting this, a recent Mendelian randomization study found that increased circulating levels of OAS1 were associated with reduced risk of very severe COVID-19, hospitalization for COVID-19 and susceptibility to this disease^9^. However, other evidence from a transcriptome-wide association study suggested a stronger association with OAS3 levels^3^. Thus, efforts are required to disentangle the causal gene, or genes, at this locus.

**Fig. 1:**
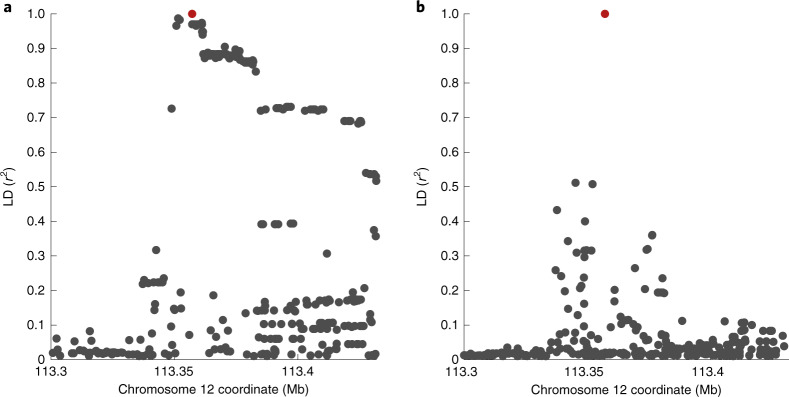
LD of the splice acceptor variant in individuals of European and African ancestries. **a**, Plot of LD in individuals of European ancestry (*n* = 503) shows that 130 variants are in LD (*r*^2^ > 0.8) with the splice acceptor variant rs10774671 (marked in red). **b**, Same as in **a** but for individuals of African ancestry (*n* = 661). No variants were found to be in LD with the splice acceptor variant. Data are from the 1000 Genomes Project^11^. The *x* axis shows the hg19 coordinates. Source data

The *OAS* region was identified as a COVID-19 risk locus in association studies^3,5^ of mainly individuals of European ancestry. The protective haplotype derived from Neanderthals in individuals of European ancestry is approximately 75 kb and spans the three genes *OAS1*, *OAS2* and *OAS3* (ref. ^7^). A candidate causal variant in the region is rs10774671, which falls in a splice acceptor site at exon 7 of *OAS1* and where the protective (G) allele results in a longer and approximately 60% more active OAS1 enzyme^10^. However, this variant is as associated with COVID-19 severity as many of the hundreds of variants in LD. For example, in individuals of European ancestry, we found 130 variants in strong LD (*r*^2^ > 0.8) with the splice acceptor variant (Fig. 1a). Thus, further methods are required to disentangle the causal SNP(s) at this locus, which could help identify the causal gene.

One method to better identify causal SNPs at an associated locus is to test associations in different ancestry groups, particularly when these other populations have different LD structures and shorter haplotypes. Therefore, to examine whether we could identify a population with which we could test this variant independently, we investigated the presence of co-segregating variants in populations in the 1000 Genomes Project^11^. In individuals of South Asian ancestry, there are 129 such variants, and in individuals of East Asian ancestry, 128 such variants exist. In stark contrast, no variants co-segregate with rs10774671 in individuals of African ancestry at an LD of *r*^2^ > 0.6 (Fig. 1b). Thus, populations of African ancestry offer a possibility to independently test whether rs10774671 is associated with COVID-19 severity. We note that the use of a reference panel such as the 1000 Genomes Project does not provide a complete picture of the LD structure of this genomic region for different ancestries. Nevertheless, it is clear that the Neanderthal haplotype is virtually absent among individuals of primarily African ancestry.

**Fig. 2:**
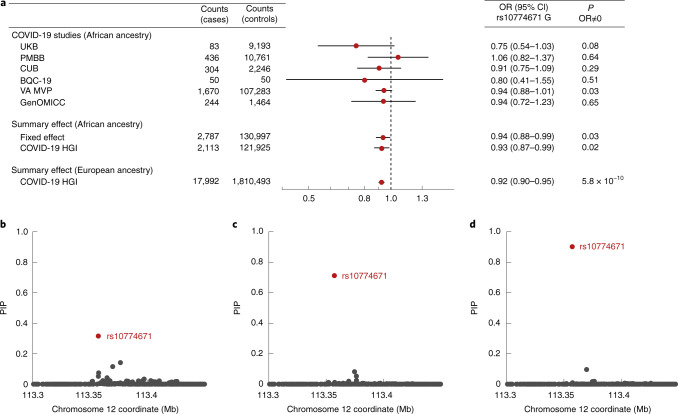
Ancestral splice variant and likelihood of hospitalization on SARS-CoV-2 infection. **a**, ORs for COVID-19 hospitalization for carriers of the ancestral splice variant of African ancestry. The plots show the summary effect in individuals of African ancestry (*n* = 2,787 cases) by meta-analysis of 6 cohorts shown in this study and by the COVID-19 HGI (*n* = 2,133 cases). Data are presented as ORs ± 95% CIs. **b**, PIPs using the summary statistics and LD from individuals of European ancestry. **c**, Same as **b** but for individuals of African ancestry, using the European PIPs as prior probabilities. **d**, Same as **c** but using scaled CADD-scores as prior probabilities for the first (European) step in the fine-mapping analysis. The error bars in **a** show the 95% CIs. The *x* axes in **b**–**d** show the genomic coordinates in the hg19.

To test the association of splice acceptor variant rs10774671 with COVID-19 outcomes in people of African ancestry, we combined six studies that had assessed COVID-19 severity (UK Biobank (UKB), Penn Medicine BioBank (PMBB), Columbia University Biobank (CUB), Biobanque Québécoise de la COVID-19 (BQC-19), GenOMICC and the VA Million Veteran Program (MVP)), comprising 2,787 cases and 130,997 controls of African ancestry. We found that the rs10774671 G allele conferred protection against COVID-19 hospitalization in individuals of African ancestry (Fig. 2a, *P* = 0.03) of similar magnitude (odds ratio (OR) = 0.94, 95% confidence interval (CI) = 0.88–0.99) as in individuals of European ancestry (OR = 0.92, 95% CI = 0.90–0.95), in whom the rs10774671 G allele is less common (32% allele frequency among individuals of European ancestry versus 58% among individuals of African ancestry). We found no evidence of heterogeneity across the 5 studies (Cochran’s *Q* = 3.22, *P* = 0.67; *I*^2^ = 0% (0.0–74.6%); *τ*^2^ = 0 (0–0.05), 95% CI in brackets; Methods), and there was no statistical difference (*P* = 0.72) between the 3 cohorts that used methods that specifically control for unbalanced case–control ratio (that is, regenie or the Scalable and Accurate Implementation of GEneralized mixed model (SAIGE)) and the 3 that did not (Methods). Moreover, a meta-analysis of overlapping individuals of African ancestry performed by the COVID-19 Host Genetics Initiative^5^ (HGI) yielded similar results (OR = 0.93, 95% CI = 0.87–0.99, *P* = 0.02; 2,113 cases and 121,925 controls). Thus, the rs10774671 G allele confers protection against COVID-19 severity independently of the variants with which it is associated in non-African populations.

Although we found nominal statistical support (HGI one-tailed *P* = 9.0 × 10^−3^) for the hypothesis of an effect in the same direction for the splice variant among individuals of African ancestry, this association alone does not rule out other causal variants. To test the splice acceptor variant rs10774671 against other candidate variants, we performed stepwise fine-mapping using individuals of both European and African ancestry. Summary statistics and LD for individuals of European ancestry could not resolve the association signal at this locus (Fig. 2b, posterior inclusion probability (PIP) for rs10774671 = 0.31). Using the PIPs from this analysis as prior probabilities when analyzing African ancestry data identified rs10774671 as most likely to be causal (Fig. 2c, PIP = 0.71). Combining both ancestries with in silico prediction of deleteriousness (CADD-scores^12^, which exist for both synonymous and nonsynonymous variants) brings further support that rs10774671 is the causal variant (Fig. 2d, PIP = 0.90). We also find that one causal variant is more likely than two (*P* = 0.86 versus *P* = 0.14).

This observation is compatible with the fact that Neanderthal haplotypes are rare or absent in African populations^13,14^ and that ancestral alleles seen in Neanderthals, such as the G allele at rs10774671, exist today as a result of their inheritance from the ancestral population common to both modern humans and Neanderthals. In the latter case, such variants have existed in modern humans on the order of approximately half a million years and therefore co-segregate with different variants than when they are derived from gene flow from Neanderthals into modern humans that occurred about 60,000 years ago^15^. In this study, we leverage this fact to show that the ancestral splice variant, encoding a more active^10^ and prenylated form of OAS1 with capacity for membrane localization^16^, is responsible for the protective effect associated with this locus^17^. These findings provide evidence that the splice site variant at this locus influences COVID-19 outcomes by altering the splicing of *OAS1*. Furthermore, this insight highlights the importance of including populations of different ancestries in genetic association studies and rapidly sharing data through large, international consortia.

## Methods

### Ethics

This study complies with all relevant ethical regulations; the contributing genetic association studies were approved by the VA Central institutional review board (VA MVP), the Jewish General Hospital research ethics board (BQC-19), the institutional review board of the Perelman School of Medicine at the University of Pennsylvania (PMBB), the institutional review board of Columbia University (CUB), the research ethics committees of Scotland (15/SS/0110), England, Wales and Northern Ireland (19/WM/0247) (GenOMICC) and the North West Multi-Centre Research Ethics Committee (UKB).

### Study participants

Our analysis pooled hospitalized patients with COVID-19 of African ancestry (*n* = 2,787) from 6 cohorts. The UKB cohort contained 83 cases and 9,193 controls, the PMBB contained 436 cases and 10,761 controls, the CUB contained 304 cases and 2,246 controls, BQC-19 contained 50 cases and 50 controls, VA MVP contained 1,670 cases and 107,283 controls and GenOMICC contained 244 cases and 1,464 controls. Informed consent was obtained when required and ethical approval was obtained from the relevant research ethics boards. Ancestry was genetically inferred (see below for each cohort).

### LD

LD (*r*^2^) was calculated using LDlink^18^ v.4.1 in the genomic region 113.30–113.45 megabases (Mb) (hg19) using data from the 1000 Genomes Project^11^.

### Meta-analysis

The meta-analysis was done using inverse variance weighting in the R package meta v.5.1. Heterogeneity was measured using Cochran’s *Q*, Higgins–Thompson’s *I*^2^ and *τ*^2^ using the DerSimonian–Laird estimator.

### Fine-mapping

Effect estimates and standard errors in the genomic region 113.30–113.45 Mb were taken from the HGI^5^ for individuals of European and African ancestry. LD (*r*^2^) was obtained from the 1000 Genomes Project^11^. Fine-mapping with shotgun stochastic search was performed using FINEMAP v.1.4 (ref. ^17^) with effect size estimates in the region for both ancestries (*n* = 383), excluding one triallelic variant (rs1051042). Importantly, among the 383 variants in the fine-mapping analysis, all 131 variants with genome-wide significance (*P* < 5 × 10^−8^) among individuals of European ancestry were included. First, the European ancestry data were fine-mapped using the equal probabilities of each variant as prior probabilities. The PIPs of this analysis were then used as prior probabilities in an analysis of the African ancestry data. Finally, this stepwise analysis was repeated but using scaled CADD-scores^11^ v.1.6 as prior probabilities for the first (European ancestry) step. The prior probabilities based on the CADD-scores were normalized so that the sum equaled 1. The variance of the effect size prior was set such that with 95% probability a variant can increase risk by at most an OR of 2 and then scaled using the case–control ratio as described^19^.

### VA MVP summary statistics

The VA MVP is a US-based longitudinal research program investigating how genes, lifestyle and military exposure influence health and illness in veterans, with study recruitment commencing in 2011 (ref. ^20^). Study participants were genotyped using a customized Affymetrix Axiom Biobank Array (the MVP v.1.0 Genotyping Array) containing 723,305 variants^21^. Imputation was performed to a hybrid imputation panel consisting of the African Genome Resources panel (https://imputation.sanger.ac.uk/?about=1#referencepanels) and 1000 Genomes Project v3p5. COVID-19 cases were identified using an algorithm developed by the VA COVID National Surveillance Tool^22^. COVID-19-related hospitalizations were defined as hospital admissions between 7 d before and 30 d after an individual’s positive SARS-CoV-2 test. The association of hospitalized cases with COVID-19 versus all other MVP participants was tested under an additive logistic model and was corrected for age, age^2^, sex, age-by-sex and ethnicity-specific principal components. Individuals who died before 1 March 2020 were excluded, as was one individual from each related pair. The analysis was restricted to only African American MVP participants (as defined by HARE^23^), resulting in 1,300 cases and 98,129 controls.

### BQC-19 summary statistics

The BQC-19 is a prospective hospital-based biobank recruiting patients with proven or suspected COVID-19 (institutional review board no. 2020–2137). Whole-genome genotyping was performed for all participants, with imputation using the TOPMed Imputation Server^24^. Individuals of African ancestry were determined by projecting genetic principal components on the 1000 Genomes Project reference panel. Cases (*n* = 50) were defined as patients hospitalized with COVID-19 or who died from the infection. Controls (*n* = 50) were other participants of African ancestry, of which 32 had a clinical presentation consistent with COVID-19 but never had a positive test. An additive logistic regression model with the first ten genetic principal components, with age, sex, age^2^, age-by-sex and age^2^-by-sex as covariates, was used to determine the effect of the protective G allele on the risk of being a case.

### PMBB summary statistics

The PMBB contains approximately 60,000 prospectively consented participants, all patients of the Penn Medicine hospitals, for whom DNA samples were obtained and on whom extensive phenotypic information was generated from the electronic health record (EHR). A total of 20,079 participants were genotyped using the Illumina Global Screening Array v.2.0 and further imputed using the TOPMed Imputation Server. SNPs with a call rate <1%, minor allele frequency (MAF) <1% or imputation info score <0.3 were excluded from further analysis. To define each ancestral group, principal component analysis (PCA) was performed after merging the PMBB data with the 1000 Genomes Project reference dataset using the smartpca module of the Eigensoft package (version 7.2)^25,26^. We performed quantitative discriminant analysis on all samples using the 1000 Genomes Project samples as a training sets to generate ancestry calls for all PMBB samples included in the analysis. Ultimately 9,015 African ancestry genotyped samples were identified and included in our association study. All PMBB participants were followed for SARS-CoV-2 infection and hospitalization, with COVID-19 infection defined as any patient with a positive SARS-CoV-2 nasal swab or for whom the International Classification of Diseases billing code U07.1 was coded in the EHR and with COVID-19-related hospitalizations defined as the subset of these patients who had been admitted to hospital in the previous year with U07.1 as the admission diagnosis code or who had been admitted for COVID-19-related symptoms as determined by manual chart review. Association analyses were performed using the Firth logistic regression test as implemented in regenie^27^, including age, age^2^, sex, age-by-sex and the first six ancestry-specific principal components of the genomic data as covariates.

### CUB summary statistics

The COVID-19 CUB was established in response to the New York City infection surge in March 2020. The biobank recruited COVID-19 cases of diverse ancestry among all patients who were treated at Columbia University Irving Medical Center between March and May 2020. All cases were diagnosed by positive SARS-CoV-2 PCR test based on nasopharyngeal samples. The mean age of cases was 62.9 years and the percentage of females was 43%. The DNA of whole-blood samples was extracted using standard procedures and genotyping was performed using the Illumina Global Diversity Array chip. The controls were genotyped using the Illumina Multi-Ethnic Global Ancestry chip. The analysis of intensity clusters and genotype calls was performed with the Illumina Genome Studio software (version 2.0); all SNPs were called on forward DNA strand and standard quality control filters were applied, including a per-SNP genotyping rate >95%, per-individual genotyping rate >90%, MAF > 0.01, and Hardy–Weinberg equilibrium test *P* > 10^−8^ in controls. The duplicates and cryptic relatedness in the given cohort were determined and excluded based on an estimated pairwise kinship coefficient >0.0884. After quality control, the dataset consisted of 6,757 individuals (1,029 cases and 5,728 controls) genotyped for 1,096,321 SNPs with an overall genotyping rate of 99.9%. The imputation analysis was performed with the TOPMed Imputation Server. A total of 13,439,413 common markers imputed at high quality (*r*^2^ > 0.8 and MAF > 0.01) were used in the downstream analyses. To define the African ancestry cluster, we used PCA against the 1000 Genomes Project reference populations followed by *k*-means clustering on significant principal components of ancestry. The African ancestry cluster contained 332 cases positive for SARS-CoV-2 and 2,246 population controls. Of the 332 cases of African ancestry, 304 had severe COVID-19 requiring hospitalization. Among the 304 cases included, 78 (26%) had respiratory failure requiring intubation and invasive ventilatory support and 86 (28%) died due to COVID-19. We then tested the effect of rs10774671 G on the risk of hospitalization using SAIGE^28^, after adjustment for sex and five principal components of ancestry. The collection of samples was approved under institutional review board protocol no. AAAS7370, while the genetic analyses were approved under institutional review board protocol no. AAAS7948.

### GenOMICC summary statistics

A total of 3,893 critically ill cases with confirmed COVID-19 were recruited through the GenOMICC study in 208 intensive care units across the UK; 682 additional hospitalized cases with confirmed COVID-19 were recruited through the International Severe Acute Respiratory Infection Consortium (Coronavirus Clinical Characterisation Consortium). Current and previous versions of the study protocol are available at https://genomicc.org/protocol/. All participants gave informed consent (https://genomicc.org/protocol/#informed-consent). DNA extraction, sample quality control, genotype quality control, kinship estimation and imputation were performed with the pipelines described in Pairo-Castineira et al.^3^. Ancestry was inferred using ADMIXTURE and the superpopulations defined in the 1000 Genomes Project (European, South Asian, East Asian, African and American). When one individual had a probability >80% of pertaining to one ancestry, they were assigned to this ancestry; otherwise, they were assigned to the ‘admixed’ ancestry. After these steps, there were 244 unrelated individuals of African ancestry. Principal components were calculated according to the procedure outlined in Pairo-Castineira et al.^3^ for GenOMICC participants and UKB individuals. UKB participants were considered as potential controls if they were not identified by the UKB as outliers based on either genotyping missingness rate or heterogeneity; their sex was inferred from the genotypes that matched their self-reported sex. After excluding participants who had received PCR tests for COVID-19, based on the information downloaded from the UKB in August 2020, five random UKB individuals with matching inferred ancestry were sampled for each GenOMICC participant as controls. After sampling each control, individuals related up to the third degree were removed from the pool of potential further controls. Test for association between case–control status and allele dosage at the variant rs10774671 G was performed by fitting a logistic regression model using PLINK v.2.00 with sex, age, mean-centered age^2^, deprivation score decile of residential postcode and the first 10 genomic principal components as covariates. This research was conducted using the UKB resource under project no. 788.

### UKB summary statistics

Association analyses were performed using the Firth logistic regression test implemented in regenie, including age, age^2^, sex, age-by-sex, age^2^-by-sex and ten ancestry-informative principle components as covariates. Data were downloaded from https://rgc-covid19.regeneron.com/results (23 March 2021). Continental ancestries were determined by projecting each sample onto principal components calculated from the HapMap3 reference panel, followed by kernel density estimation yielding a likelihood that a given sample belonged to each continental ancestry, as described previously^29^.

### Reporting Summary

Further information on research design is available in the Nature Research Reporting Summary linked to this article.

## Online content

Any methods, additional references, Nature Research reporting summaries, source data, extended data, supplementary information, acknowledgements, peer review information; details of author contributions and competing interests; and statements of data and code availability are available at 10.1038/s41588-021-00996-8.

## Supplementary information

Reporting Summary

Peer Review Information

## Data Availability

COVID-19 summary statistics for individuals of African ancestry are available at https://www.covid19hg.org/results/r6/ and CADD-scores v.1.6 can be accessed at https://cadd.gs.washington.edu/score. Genomes from the 1000 Genomes Project are available at https://www.internationalgenome.org/data. The fine-mapping association summary statistics produced in this study are available at 10.5281/zenodo.5708333. Source data are provided with this paper.

## Source data

Source Data Fig. 1 LD in the *OAS1/2/3* region.

## Code availability

The code used for the fine-mapping analysis can be found at 10.5281/zenodo.5708333.
