# Supplementary material for: Multi-ancestry fine mapping implicates OAS1 splicing in risk of severe COVID-19
Source: Nat Genet. 2022 Jan 13;54(2):125–7. doi: 10.1038/s41588-021-00996-8 (PMC8837537; doi:10.1038/s41588-021-00996-8)
Supplement: Supplementary file 1 — Reporting Summary [file 41588_2021_996_MOESM1_ESM.pdf]

nature research

Corresponding author(s): Hugo Zeberg

Last updated by author(s): Nov 19, 2021

# Reporting Summary

Nature Research wishes to improve the reproducibility of the work that we publish. This form provides structure for consistency and transparency in reporting. For further information on Nature Research policies, see our Editorial Policies and the Editorial Policy Checklist.

## Statistics

For all statistical analyses, confirm that the following items are present in the figure legend, table legend, main text, or Methods section.

| n/a | Confirmed | |
|---|---|---|
| ☐ | ☒ | The exact sample size (*n*) for each experimental group/condition, given as a discrete number and unit of measurement |
| ☐ | ☒ | A statement on whether measurements were taken from distinct samples or whether the same sample was measured repeatedly |
| ☐ | ☒ | The statistical test(s) used AND whether they are one- or two-sided<br>*Only common tests should be described solely by name; describe more complex techniques in the Methods section.* |
| ☐ | ☒ | A description of all covariates tested |
| ☐ | ☒ | A description of any assumptions or corrections, such as tests of normality and adjustment for multiple comparisons |
| ☐ | ☒ | A full description of the statistical parameters including central tendency (e.g. means) or other basic estimates (e.g. regression coefficient) AND variation (e.g. standard deviation) or associated estimates of uncertainty (e.g. confidence intervals) |
| ☐ | ☒ | For null hypothesis testing, the test statistic (e.g. *F*, *t*, *r*) with confidence intervals, effect sizes, degrees of freedom and *P* value noted<br>*Give P values as exact values whenever suitable.* |
| ☒ | ☐ | For Bayesian analysis, information on the choice of priors and Markov chain Monte Carlo settings |
| ☒ | ☐ | For hierarchical and complex designs, identification of the appropriate level for tests and full reporting of outcomes |
| ☒ | ☐ | Estimates of effect sizes (e.g. Cohen's *d*, Pearson's *r*), indicating how they were calculated |

*Our web collection on statistics for biologists contains articles on many of the points above.*

## Software and code

Policy information about availability of computer code

| | |
|---|---|
| Data collection | The summary statistics of each cohort was provided as described in the Methods section. |
| Data analysis | The meta-analysis of the summary statistics was done in R using the package 'meta' version 5.1. Linkage disequilibrium was calculated using LDlink version 4.1. Fine mapping was performed using FINEMAP version 1.4. Custom code to incorporate prior probabilites in the fine-mapping analysis is deposited at https://doi.org/10.5281/zenodo.5708333. |

For manuscripts utilizing custom algorithms or software that are central to the research but not yet described in published literature, software must be made available to editors and reviewers. We strongly encourage code deposition in a community repository (e.g. GitHub). See the Nature Research guidelines for submitting code & software for further information.

## Data

Policy information about availability of data

All manuscripts must include a data availability statement. This statement should provide the following information, where applicable:

- Accession codes, unique identifiers, or web links for publicly available datasets
- A list of figures that have associated raw data
- A description of any restrictions on data availability

COVID-19 summary statistics for African ancestry and European ancestry individuals are available at https://www.covid19hg.org/results/r6/. CADD-scores (version 1.6) can be accessed at https://cadd.gs.washington.edu/score. Genomes from the 1000 Genomes Project are available at https://www.internationalgenome.org/data. Fine-mapping association summary statistics produced in this study are availabe at https://doi.org/10.5281/zenodo.5708333.

# Field-specific reporting

Please select the one below that is the best fit for your research. If you are not sure, read the appropriate sections before making your selection.

☒ Life sciences ☐ Behavioural & social sciences ☐ Ecological, evolutionary & environmental sciences

For a reference copy of the document with all sections, see nature.com/documents/nr-reporting-summary-flat.pdf

# Life sciences study design

All studies must disclose on these points even when the disclosure is negative.

| | |
|---|---|
| Sample size | All available data on COVID-19 hospitalziation in Africans were used. |
| Data exclusions | Where appropriate, related individuals were excluded to avoid genetic biases. This was done lege artis and not post-hoc. |
| Replication | The direction of the effect was seen in 4 out 5 cohorts. We could not detect any heterogeneity accross the cohorts. |
| Randomization | Not applicable for a genetic association study of this kind, more than the stochastic nature of whom that get infected. It would generally be unethical to randomize individuals to get deliberately infected with SARS-CoV-2. |
| Blinding | Not applicable for a genetic association study of this kind. The medical staff could not be blinded to the patients' state of health. The genotype status did not influence the choice to hospitalize COIVD-19 patients. The case status and severity of symptoms was evaluated for each sample by investigators from each study respectively. |

# Reporting for specific materials, systems and methods

We require information from authors about some types of materials, experimental systems and methods used in many studies. Here, indicate whether each material, system or method listed is relevant to your study. If you are not sure if a list item applies to your research, read the appropriate section before selecting a response.

## Materials & experimental systems

| n/a | Involved in the study | |
|---|---|---|
| ☒ | ☐ | Antibodies |
| ☒ | ☐ | Eukaryotic cell lines |
| ☒ | ☐ | Palaeontology and archaeology |
| ☒ | ☐ | Animals and other organisms |
| ☐ | ☒ | Human research participants |
| ☒ | ☐ | Clinical data |
| ☒ | ☐ | Dual use research of concern |

## Methods

| n/a | Involved in the study | |
|---|---|---|
| ☒ | ☐ | ChIP-seq |
| ☒ | ☐ | Flow cytometry |
| ☒ | ☐ | MRI-based neuroimaging |

## Human research participants

Policy information about studies involving human research participants

| | |
|---|---|
| Population characteristics | Hospitalized COVID-19 positive individuals of Afrian (n = 2,787) and European (n = 17,992) ancestries. The mean age of cases across the studies was 55.3 years (as reported by the COVID19 Host Genetics Intitative in the flagship paper: doi: https://doi.org/10.1038/s41586-021-03767-x). Population characteristics for the contributing studies to the COVID19 Host Genetics Intitative are given in Supplementary Table 1 of the flagship paper. |
| Recruitment | Depending on cohort, participants were either reqruited upon infection or were already included in a prospective longitudinal cohort. |
| Ethics oversight | This study complies with all relevant ethical regulations and the contributing genetic association studies were approved by the VA Central Institutional Review Board (VA Million Veteran Program), the Jewish General Hospital research ethics board (Biobanque Québécoise de la COVID-19), the Institutional Review Board of Perelman School of Medicine at University of Pennsylvania (Penn Medicine Biobank), the Institutional Review Board of Columbia University (Columbia University Biobank), the research ethics committees of Scotland 15/SS/0110; England, Wales and Northern Ireland 19/WM/0247 (GenOMICC), and the North West Multi-centre Research Ethics Committee (UK Biobank). |

Note that full information on the approval of the study protocol must also be provided in the manuscript.
