# Supplementary material for: Multi-ancestry fine mapping implicates OAS1 splicing in risk of severe COVID-19
Source: Nat Genet. 2022 Jan 13;54(2):125–7. doi: 10.1038/s41588-021-00996-8 (PMC8837537; doi:10.1038/s41588-021-00996-8)
Supplement: Supplementary file 2 — Peer Review Information [file 41588_2021_996_MOESM2_ESM.pdf]

natureresearch

Peer Review Information

Journal: Nature Genetics
Manuscript Title: Multi-ancestry fine mapping implicates OAS1 splicing in risk of severe COVID-19
Corresponding author name(s): Dr Hugo Zeberg

Reviewer Comments & Decisions:

Decision Letter, initial version:

16th April 2021

Dear Hugo,

Your Brief Communication entitled "Alternative splicing of OAS1 alters the risk for severe COVID-19" has been seen by three referees, whose comments are copied below. While they find your work of potential interest, they have raised substantial concerns that in our view are sufficiently important that they preclude publication of the work in Nature Genetics, at least in its present form.

In particular, while the referees appreciate the importance of the topic, they raise overlapping concerns about the strength of the statistical support for the association signal at rs10774671 (which could potentially be addressed by collaborating with other groups to increase the sample size) and the lack of comprehensive analysis of other variants in this region (which could be addressed by extending the analyses along the lines suggested by the referees).

Should additional data and analyses allow you to fully address these concerns, we would be willing to consider an appeal of our decision (unless, of course, something similar has by then been accepted at Nature Genetics or appeared elsewhere). This includes submission or publication of a portion of this work someplace else.

We hope you understand that until we have read the revised manuscript in its entirety we cannot promise that it will be sent back for peer review.

If you are interested in attempting to revise this manuscript for submission to Nature Genetics in the future, please contact me to discuss a potential appeal. Otherwise, we hope that you find our referees' comments helpful when preparing your manuscript for resubmission elsewhere.

Sincerely,
Kyle

Kyle Vogan, PhD
Senior Editor
Nature Genetics
https://orcid.org/0000-0001-9565-9665

Referee expertise:

Referee #1: Population genetics, statistical methods

Referee #2: Population genetics, genetic epidemiology

Referee #3: Genetics, populations of African ancestry

Reviewers' Comments:

Reviewer #1:
Remarks to the Author:

Huffman et al. use data from a number of biobanks to assess associations with OAS1 with an eye towards associations in African-descent populations. This is important to furthering analyses in Covid given how African populations, and populations of the African diaspora, are highly under-represented in modern genomic studies. However in this instance, the overall statistical support is underwhelming and the archaic introgression piece is not particularly relevant to the disease association of interest.

Major concerns: the discussion of overall association is not very strong, across all their datasets having a p=0.03 is not strong evidence, even at a candidate locus. The integration of datasets not used in the COVID HGI is important but likely there are other, more important loci given variants seen previously. Further, the question of commonality of the rs10774671 allele is a bit out of place when the allele frequencies are almost flipped in African-descent and European-descent populations, the power should be pretty similar if the AFs are 66% and 35% respectively.

Minor points:
Abstract

Specify Chromosomal region instead of 'A locus'
State odds ratio or relative risk percentage instead of 'a magnitude similar to'

Main Text
Instead of 'a longer and more active OAS1 enzyme' specify the specific exonic change and/or how much more active – from citation [9]

When mentioning case/control numbers and imbalance it is worth mentioning the use of SAIGE, it's

mentioned later in the methods, but just as an assurance against the imbalance miscalibrating association statistics.

When mentioning ‘rs10774671 is associated with COVID-19 severity independently’ - the description of effect direction was a bit vague, it is worth being more precise given the different ancestries and varying allele frequency across populations.

Methods
‘pooled hospitalized COVID-19 patients of African ancestry’ - are these self-identified or derived ancestry labels?

Major comment: ‘DNA of whole blood samples was extracted using standard procedures and genotyping was performed using the Illumina Global Diversity Array (GDA) chip. The controls were genotyped using the Illumina Multiethnic Global Ancestry (MEGA) chip

Did they restrict SNP call sets to the overlap of these two chips? The call set should overlap pretty good but just want to make sure it was handled properly.

Mention SAIGE testing earlier in main text as well.

Reviewer #2:
Remarks to the Author:

Huffman at el. aimed to test for association between a potentially causal SNP, rs10774671, within the COVID severity risk locus, OAS1/2/3 (12q24.13). To do this, they examined the association between this SNP and COVID severity in five study populations of African ancestry, as LD patterns suggest that the risk SNP is independent of other nearby SNPs. They subsequently identified a significant association and conclude that this candidate SNP confers protection against COVID-19. Overall, this brief communication is well-written and the approach to confirming that this lead SNP may be the causal SNP differs from other publications. However, I have a few concerns regarding the communication of the methods and conclusions.

Major concerns:

1. The conclusion that the authors, “show that among several potentially causal variants at this locus, a splice site variant of OAS1… confers protection against COVID-19…”, is overstated. The observed association in the current study is somewhat weak (P=0.03, OR=0.92), and without examination of other nearby variants, some caution in interpretability should be provided.

2. It would help to provide a brief review of the study limitations and highlight potential next directions. This candidate variant study highlights the need for a robust examination of this locus, such as leveraging differences in ancestral LD to narrow in on causal variants covering the entire region. As the authors note in the introduction their may be more than one causal variant in this locus.

3. Related to the previous concern, the authors do not state that the SNP examined here is the lead variant identified by the previous GWASs, i.e. COVID-19 Host Genetics Initiative. Was this part of the

motivation for focusing the examination on this variant? While the SNP is a splice site acceptor, which makes it is likely candidate, as the authors note, there are several potentially causal variants in this region not examined here. Additionally, there has been mixed evidence about the causal gene in this region, as noted by the authors. Were there similarly associated, putatively causal variants within OAS3/2 that could have benefited from this study design as well?

4. The authors do not provide consistent details about study design across each site. For example, the UKBB data were analyzed using REGENIE using firth logistic regression, and Columbia used SAIGE, but software and specific assumptions and models are not provided for other studies. How ancestry was inferred across the studies is not reported by each study and may be important here.

5. Other methodological information that may help interpretation of results are missing. For example, was the variant in question imputed or genotyped? If imputed, how well imputed was the variant (especially since LD in this region for the African ancestry individuals is sparse)? Why was whole exome sequencing not used for those studies that had that available? What is the sample overlap between these studies and the published discovery analysis?

Minor Concerns

1. The sentence on page three that begins with, “In the latter case, variants have existed in modern humans in the order of half a million years ago and therefore …” seems to be missing a word or two.

2. Please add study specific MAF to Fig 2. This may be important for meta-analysis given different regression models (although unlikely given that the variant should be common across studies).

Reviewer #3:
Remarks to the Author:

It is stated in the abstract that they “show that among several potentially causal variants at this locus, a splice variant of OAS1 occurs in people of African ancestry independently of the Neanderthal haplotype and confers protection against COVID-19 of a magnitude similar to that seen in individuals without African ancestry.” This is in fact my major concern with the approach taken – I think the entire work is really only focused on the single variant (rs10774671 – a splice acceptor site) despite there being other functional variants as described in their PNAS work. There, in addition to the splice acceptor site, they report that the Neandertal haplotype contains a missense variant (rs2660) in OAS1, a missense variant (rs1859330) and two synonymous variants (rs1859329 and rs2285932) in OAS3, and a missense variant in OAS2 (rs1293767), three of which are ancestral and occur in Africa (rs2660, rs1859330, and rs1859329). It does not appear that the approach used here truly examines the variants relatively.

Fig 1A shows the LD between all other variants indexed on the splice acceptor sites, however what is the LD in the region? What is the LD across the region in general, especially between-variant LD for other putative variants listed in the prior work? A pairwise summary would be more appropriate to fully disentangle the relationship among all potentially causal variants as indicated in the abstract.

I have no concerns with the summary of the specific analysis on rs10774671 – the analysis across the

studies, the effect size, lack of heterogeneity all make a cohesive story. However, what is the association across the region in these African ancestry subjects. It is a missed opportunity first, but also an incomplete picture to limit the association analysis to only the one credible functional variant where there are others, and the argument to limit it to this based on its lack of LD to other variants in the African ancestry 1000 Genomes data is not fully understood. While other variants may not be in LD, they may potentially have stronger associations in the African ancestry sample, maybe even helping identify secondary signals that cannot be separated from this variant because of the LD in the largely European ancestry samples thus far. Along this thread, I find the conclusion that this work show that the ancestral splice variant, encoding a longer and more active enzyme, is ‘responsible’ for the protective effect associated with this locus to an overstatement of the findings of the work.

Alternative splicing of OAS1 alters the risk for severe COVID-19 : I find the title misleading as the body of this word does not in fact evaluate the functionality of the alternative splicing. Rather is using LD architecture across populations to support the association signal for this variant.

Decision Letter, first revision:

IMPORTANT: Please note the reference number: NG-BC57106R-Z Zeberg. This number must be quoted whenever you communicate with us regarding this paper.

16th July 2021

Dear Hugo,

Thank you for asking us to reconsider your manuscript "Alternative splicing of OAS1 alters the risk for severe COVID-19". I have discussed your proposed resubmission with my editorial colleagues, and we invite you to formally upload your revised manuscript for further consideration and peer review.

When preparing a revision, please ensure that it fully complies with our editorial requirements for format and style; details can be found in the Guide to Authors on our website (http://www.nature.com/ng/).

Please be sure that your manuscript is accompanied by a separate letter and point-by-point response to the original reviews detailing the changes you have made and your response to the points raised. At this stage we will need you to upload:

1) a copy of the manuscript in MS Word .docx format.

2) the Editorial Policy Checklist:
https://www.nature.com/documents/nr-editorial-policy-checklist.pdf

3) the Reporting Summary:
https://www.nature.com/documents/nr-reporting-summary.pdf
(Here you can read about the role of the Reporting Summary in reproducible science:
https://www.nature.com/news/announcement-towards-greater-reproducibility-for-life-sciences-

research-in-nature-1.22062)

Please use the link below to be taken directly to the site and view and revise your manuscript:

[REDACTED]

With best wishes,
Kyle

Kyle Vogan, PhD
Senior Editor
Nature Genetics
https://orcid.org/0000-0001-9565-9665

Author Rebuttal, first revision:

n.b. all edits highlighted in yellow in the manuscript.

We would like to thank the reviewers for their valuable comments and suggestions. Below we provide detailed responses together with the corresponding additions and revisions made to the manuscript.

Reviewer #1: *Huffman et al. use data from a number of biobanks to assess associations with OAS1 with an eye towards associations in African-descent populations. This is important to furthering analyses in Covid given how African populations, and populations of the African diaspora, are highly under-represented in modern genomic studies. However in this instance, the overall statistical support is underwhelming and the archaic introgression piece is not particularly relevant to the disease association of interest.*

Major concerns*: the discussion of overall association is not very strong, across all their datasets having a p=0.03 is not strong evidence, even at a candidate locus. The integration of datasets not used in the COVID HGI is important but likely there are other, more important loci given variants seen previously. Further, the question of commonality of the rs10774671 allele is a bit out of place when the allele frequencies are almost flipped in African-descent and European-descent populations, the power should be pretty similar if the AFs are 66% and 35% respectively.*

Response: To strengthen statistical significance of our findings we have included an additional cohort (GenOMICC) where we observed the same direction of the association. Moreover, we now incorporate the recently released (July 1st) ancestry specific meta-analysis of the COVID-19 Host Genetics Imitative (HGI). We can now present a p-value of p = 0.018 (Fig. 2A). Representatives from GenOMICC and HGI have now been included as co-authors.

To address the concern of other tentative causal variants, we have performed a trans-ancestry statistical fine-mapping of the entire OAS1/2/3 locus. We have done this using an integrative approach incorporating data from both European and African ancestries.

Fig. 2B shows the results of a fine-mapping performed using only European ancestry individuals. In European-only individuals, the splice variant rs10774671 is the lead variant, but not well supported as the causal SNP (Posterior inclusion probability [PIP] 30%). However, by incorporating African ancestry individuals, this locus resolves to clearly implicate the rs10774671 (Fig. 2C, PIP = 69%). Further, the signal is additionally improved by including in-silico prediction (CADD-scores) together with data from both ancestries (Fig. 2D, PIP = 90%), with substantially lower support for any other variant.

Minor points*: Abstract: Specify Chromosomal region instead of ‘A locus’. State odds ratio or relative risk percentage instead of ‘a magnitude similar to’*

Response: We have specified the chromosomal region (12q24.13) in the abstract. The abstract no longer contains the sentence mentioning the similar magnitude, but instead highlight the fine-mapping now performed.

Main Text: Instead of ‘a longer and more active OAS1 enzyme’ specify the specific exonic change and/or how much more active – from citation [9]

Response: We agree with the reviewer on the importance of being specific. We now specify the lost/kept exon (7) and how much more active the longer isoform is (~60%).

When mentioning case/control numbers and imbalance it is worth mentioning the use of SAIGE, it's mentioned later in the methods, but just as an assurance against the imbalance miscalibrating association statistics.

Response: Three of the now included six cohorts use methods that specifically control for unbalanced case controls ratios (UK Biobank [Regenie], Penn [Regenie], and Columbia [SAIGE]), whereas three rely on normal additive logistic regression (Quebec, MVP and GenOmicc). This is now mentioned in the main text. Meta-analysing the cohorts that use SAIGE/Regenie yields = OR = 0.92, 95% CI: [0.80- 1.06] whereas the other three give OR = 0.94, 95% CI:0.88-1.01, a difference which is not significant (p = 0.72). If anything, the OR is slightly driven downwards by the SAIGE/Regenie cohorts, demonstrating that the result is not driven by imbalanced association statistics.

When mentioning 'rs10774671 is associated with COVID-19 severity independently' - the description of effect direction was a bit vague, it is worth being more precise given the different ancestries and varying allele frequency across populations.

Response: The revised man uscript makes the effect direction clear.

***Methods**: 'pooled hospitalized COVID-19 patients of African ancestry' - are these self-identified or derived ancestry labels?*

Response: The ancestries are derived. This has now been clarified.

Major comment: 'DNA of whole blood samples was extracted using standard procedures and genotyping was performed using the Illumina Global Diversity Array (GDA) chip. The controls were genotyped using the Illumina Multiethnic Global Ancestry (MEGA) chip. Did they restrict SNP call sets to the overlap of these two chips? The call set should overlap pretty good but just want to make sure it was handled properly.

Response: For the Columbia University Biobank (which the reviewer refers to), the call set was the overlap between these two sets.

Mention SAIGE testing earlier in main text as well.

Response: The use of SAIGE is now mentioned in the main text.

***<u>Reviewer #2:</u>** Huffman at el. aimed to test for association between a potentially causal SNP, rs10774671, within the COVID severity risk locus, OAS1/2/3 (12q24.13). To do this, they examined the association between this SNP and COVID severity in five study populations of African ancestry, as LD patterns suggest that the risk SNP is independent of other nearby SNPs. They subsequently identified a significant association and conclude that this candidate SNP confers protection against COVID-19. Overall, this brief communication is well-written and the approach to confirming that this lead SNP may be the causal SNP differs from other publications. However, I have a few concerns regarding the communication of the methods and conclusions.*

***Major concerns:** 1. The conclusion that the authors, "show that among several potentially causal variants at this locus, a splice site variant of OAS1… confers protection against COVID-19…", is overstated. The observed association in the current study is somewhat weak (P=0.03, OR=0.92), and without examination of other nearby variants, some caution in interpretability should be provided.*

Response: The reviewer is correct that examination of nearby-variants were warranted. We now provide a fine-mapping analysis of the entire OAS1/2/3 locus (Fig 2B-D). We thank the reviewer for raising this question. This was a shared concerns among the three reviewers.

2. It would help to provide a brief review of the study limitations and highlight potential next directions. This candidate variant study highlights the need for a robust examination of this locus, such as leveraging differences in ancestral LD to narrow in on causal variants covering the entire region. As the authors note in the introduction their may be more than one causal variant in this locus.

Response: We now bring up the use of a reference panel such as the 1000 Genomes project as a study limitation. With the analysis of the entire locus now performed we can give some estimate on the probable number of causal alleles. There is a 89% probability of just one variant driving the signal in the region, a result which is mentioned in the revised main text.

3. Related to the previous concern, the authors do not state that the SNP examined here is the lead variant identified by the previous GWASs, i.e. COVID-19 Host Genetics Initiative. Was this part of the motivation for focusing the examination on this variant? While the SNP is a splice site acceptor, which makes it is likely candidate, as the authors note, there are several potentially causal variants in this region not examined here. Additionally, there has been mixed evidence about the causal gene in this region, as noted by the authors. Were there similarly associated, putatively causal variants within OAS3/2 that could have benefited from this study design as well?

Response: Since the initial submission, a revised meta-analysis has been released by the Covid-19 Host Genetics Initiative. The splice acceptor variant rs10774671 is now the lead variant in the European meta-analysis, but just with a very slim margin which does not allow us to dissect the causality of this allele (new Fig. 2B). However, when incorporating African ancestry data (new Fig. 2C) the locus is much resolved and clearly indicates rs10774671 as the causal allele.

4. The authors do not provide consistent details about study design across each site. For example, the UKBB data were analyzed using REGENIE using firth logistic regression, and Columbia used SAIGE, but software and specific assumptions and models are not provided for other studies. How ancestry was inferred across the studies is not reported by each study and may be important here.

Response: The studies not using SAIGE or REGENIE employed a normal additive logistic regression, using covariates as described. Ancestry was inferred genetically as described in the methods. This information was missing for the UK Biobank data and has been added in the revised manuscript.

5. Other methodological information that may help interpretation of results are missing. For example, was the variant in question imputed or genotyped? If imputed, how well imputed was the variant (especially since LD in this region for the African ancestry individuals is sparse)? Why was whole exome sequencing not used for those studies that had that available? What is the sample overlap between these studies and the published discovery analysis?

Response: This varied across the different genotyping arrays used by the six cohorts. The by far largest cohort (Millions' Veterans) typed rs10774671 directly. For the entire locus (which we now analyse) we rely on the QC performed by the COVID-19 Host Genetics Initiative. In brief, an imputation INFO>0.6 was required for each variant in each cohort. Whole exome sequencing was not used because the variant rs10774671 is a splice-acceptor variant and does not fall within an exon (even though it would probably be possible to analyse overhang given the proximity to the coding sequence), and for consistency reasons we did not want to mix exome data with array data.

Minor Concerns. *1. The sentence on page three that begins with, “In the latter case, variants have existed in modern humans in the order of half a million years ago and therefore …” seems to be missing a word or two.*

Response: This sentence has been revised.

2. Please add study specific MAF to Fig 2. This may be important for meta-analysis given different regression models (although unlikely given that the variant should be common across studies).

Response: Figure has been revised and now includes a column with the G-allele frequency at rs10774671.

Reviewer #3: *It is stated in the abstract that they “show that among several potentially causal variants at this locus, a splice variant of OAS1 occurs in people of African ancestry independently of the Neanderthal haplotype and confers protection against COVID-19 of a magnitude similar to that seen in individuals without African ancestry.” This is in fact my major concern with the approach taken – I think the entire work is really only focused on the single variant (rs10774671 – a splice acceptor site) despite there being other functional variants as described in their PNAS work. There, in addition to the splice acceptor site, they report that the Neandertal haplotype contains a missense variant (rs2660) in OAS1, a missense variant (rs1859330) and two synonymous variants (rs1859329 and rs2285932) in OAS3, and a missense variant in OAS2 (rs1293767), three of which are ancestral and occur in Africa (rs2660, rs1859330, and rs1859329). It does not appear that the approach used here truly examines the variants relatively.*

Response: We thank the reviewer for raising this concern, which was raised by all three referees. We now provide a fine-mapping analysis using data for the entire locus. The posterior probabilities (PP) for the variants highlighted by the reviewer are:

rsID	PP (EUR)	PP (EUR+AFR)	PP (EUR+AFR+CADD)
rs10774671	0.25	0.63	0.87
rs2660	0.06	0.01	0.01
rs1859330	0.01	0.08	0.03
rs1859329	0.10	0.02	0.02
rs2285932	0.01	0.00	0.00

Fig 1A shows the LD between all other variants indexed on the splice acceptor sites, however what is the LD in the region? What is the LD across the region in general, especially between-variant LD for other putative variants listed in the prior work? A pairwise summary would be more appropriate to fully disentangle the relationship among all potentially causal variants as indicated in the abstract.

Response: The fine-mapping analysis now performed use an LD-matrix across all variants the region. For reference, we give the pairwise LD for the variants highlighted by the reviewers here:

EUR:

rs10774671	1.00	0.97	0.89	0.87	0.72
rs2660	0.97	1.00	0.88	0.90	0.75
rs1859330	0.89	0.88	1.00	0.98	0.79
rs1859329	0.87	0.90	0.99	1.00	0.81
rs2285932	0.72	0.75	0.79	0.81	1.00

AFR:

rs10774671	1.00	0.19	0.54	0.14	0.12
rs2660	0.19	1.00	0.23	0.86	0.75
rs1859330	0.54	0.22	1.00	0.28	0.23
rs1859329	0.14	0.86	0.28	1.00	0.85
rs2285932	0.12	0.75	0.23	0.85	1.00

I have no concerns with the summary of the specific analysis on rs10774671 – the analysis across the studies, the effect size, lack of heterogeneity all make a cohesive story. However, what is the association across the region in these African ancestry subjects. It is a missed opportunity first, but also an incomplete picture to limit the association analysis to only the one credible functional variant where there are others, and the argument to limit it to this based on its lack of LD to other variants in the African ancestry 1000 Genomes data is not fully understood. While other variants may not be in LD, they may potentially have stronger associations in the African ancestry sample, maybe even helping identify secondary signals that cannot be separated from this variant because of the LD in the largely European ancestry samples thus far. Along this thread, I find the conclusion that this work show that the ancestral splice variant, encoding a longer and more active enzyme, is 'responsible' for the protective effect associated with this locus to an overstatement of the findings of the work.

Response: We hope that the analysis of the entire locus (Fig 2B-D) now performed takes care of the reviewers concern. We appreciate that the reviewer find the story regarding rs10774671 cohesive.

Alternative splicing of OAS1 alters the risk for severe COVID-19 : I find the title misleading as the body of this word does not in fact evaluate the functionality of the alternative splicing. Rather is using LD architecture across populations to support the association signal for this variant.

Response: There is overwhelming support that the splice acceptor variant rs10774671 causes alternative splicing of the OAS1 transcript (e.g., GTEx p = 6.6e-227). We agree with the reviewer that it is a logical step to implicate the alternative transcript but we believe there is sufficient data to support this claim.

Decision Letter, second revision:

Our ref: NG-BC57106R1

19th October 2021

Dear Hugo,

Your revised manuscript "Alternative splicing of OAS1 alters the risk for severe COVID-19" (NG-BC57106R1) has been seen by two of the original referees. As you will see from their comments below, they find that the paper has improved in revision, and therefore we will be happy in principle to publish it in Nature Genetics as a Brief Communication pending final revisions to satisfy Reviewer #2's remaining requests and to comply with our editorial and formatting guidelines.

We are now performing detailed checks on your paper and we will send you a checklist detailing our editorial and formatting requirements soon. Please do not upload the final materials and make any revisions until you receive this additional information from us.

Thank you again for your interest in Nature Genetics. Please do not hesitate to contact me if you have any questions.

Sincerely,
Kyle

Kyle Vogan, PhD
Senior Editor
Nature Genetics
https://orcid.org/0000-0001-9565-9665

Reviewer #2 (Remarks to the Author):

Huffman et al. have examined the OAS1/2/3 COVID-19 severity locus to narrow in on causal variants. In response to previous critiques, they have clarified details of the study design, and importantly, added fine-mapping of the association region. The additional results and analyses presented in this revision have improved the presentation and confidence in the results. I have a few remaining minor concerns.

1. The title is still somewhat misleading. As other reviewers also pointed out, this work does not evaluate the functionality of the alternative splicing variant directly but attempts to identify an association in African ancestry individuals and leverage LD and annotation to support the causality of this variant statistically. Perhaps something that speaks more to the analyses conducted directly may be better suited, in other words referencing the authors' use of ancestrally diverse samples to narrow in on causal variants increasing risk for severe COVID-19 at the OAS1 locus.

2. Please clarify how many variants across what range were investigated in the fine-mapping analysis (383 (line 196) or 131 (line 197)).

3. Age2 should be age^2 across the methods.

4. The first line of “Summary statistics - UK BioBank” is repeated.

Reviewer #3 (Remarks to the Author):

My prior concerns were suitably addressed.

Author Rebuttal, second revision:

We would like to thank the reviewers for their valuable comments and suggestions. Below we provide detailed responses together with the corresponding additions and revisions made to the manuscript.

<u>Reviewer #2:</u> *Huffman et al. have examined the OAS1/2/3 COVID-19 severity locus to narrow in on causal variants. In response to previous critiques, they have clarified details of the study design, and importantly, added fine-mapping of the association region. The additional results and analyses presented in this revision have improved the presentation and confidence in the results. I have a few remaining minor concerns.*

1. *The title is still somewhat misleading. As other reviewers also pointed out, this work does not evaluate the functionality of the alternative splicing variant directly but attempts to identify an association in African ancestry individuals and leverage LD and annotation to support the causality of this variant statistically. Perhaps something that speaks more to the analyses conducted directly may be better suited, in other words referencing the authors' use of ancestrally diverse samples to narrow in on causal variants increasing risk for severe COVID-19 at the OAS1 locus.*

Response: We have changed the title to "Multi-ancestry fine mapping implicates OAS1 splicing in risk of severe COVID-19" to address this concern.

2. *Please clarify how many variants across what range were investigated in the fine-mapping analysis (383 (line 196) or 131 (line 197)).*

Response: We have clarified this. All 383 variants are included in the analysis, which includes (as a subset) all 131 variants which are genome-wide significant among Europeans.

3. *Age2 should be age^2 across the methods.*

Response: We apologize for these typos which have been corrected.

3. *The first line of "Summary statistics - UK BioBank" is repeated.*

Response: The repeated sentence has been removed.

Reviewer #3 (Remarks to the Author): *My prior concerns were suitably addressed.*

Final Decision Letter:

In reply please quote: NG-BC57106R2 Zeberg

29th November 2021

Dear Hugo,

I am delighted to say that your manuscript "Multi-ancestry fine mapping implicates OAS1 splicing in risk of severe COVID-19" has been accepted for publication in an upcoming issue of Nature Genetics.

Over the next few weeks, your paper will be copyedited to ensure that it conforms to Nature Genetics style. Once your paper is typeset, you will receive an email with a link to choose the appropriate publishing options for your paper and our Author Services team will be in touch regarding any additional information that may be required.

After the grant of rights is completed, you will receive a link to your electronic proof via email with a request to make any corrections within 48 hours. If, when you receive your proof, you cannot meet this deadline, please inform us at rjsproduction@springernature.com immediately.

You will not receive your proofs until the publishing agreement has been received through our system.

Due to the importance of these deadlines, we ask you please us know now whether you will be difficult to contact over the next month. If this is the case, we ask you provide us with the contact information (email, phone and fax) of someone who will be able to check the proofs on your behalf, and who will be available to address any last-minute problems.

Your paper will be published online after we receive your corrections and will appear in print in the next available issue. You can find out your date of online publication by contacting the Nature Press Office (press@nature.com) after sending your e-proof corrections. Now is the time to inform your Public Relations or Press Office about your paper, as they might be interested in promoting its publication. This will allow them time to prepare an accurate and satisfactory press release. Include your manuscript tracking number (NG-BC57106R2) and the name of the journal, which they will need when they contact our Press Office.

Before your paper is published online, we will be distributing a press release to news organizations worldwide, which may very well include details of your work. We are happy for your institution or funding agency to prepare its own press release, but it must mention the embargo date and Nature Genetics. Our Press Office may contact you closer to the time of publication, but if you or your Press Office have any enquiries in the meantime, please contact press@nature.com.

Acceptance is conditional on the data in the manuscript not being published elsewhere, or announced in the print or electronic media, until the embargo/publication date. These restrictions are not intended to deter you from presenting your data at academic meetings and conferences, but any enquiries from the media about papers not yet scheduled for publication should be referred to us.

Please note that Nature Genetics is a Transformative Journal (TJ). Authors may publish their research with us through the traditional subscription access route or make their paper immediately open access through payment of an article-processing charge (APC). Authors will not be required to make a final decision about access to their article until it has been accepted. Find out more about Transformative Journals

<B>Authors may need to take specific actions to achieve compliance with funder and institutional open access mandates. For submissions from January 2021, if your research is supported by a funder that requires immediate open access (e.g. according to Plan S principles), then you should select the gold OA route, and we will direct you to the compliant route where possible. For authors selecting the subscription publication route, our standard licensing terms will need to be accepted, including our self-archiving policies. Those standard licensing terms will supersede any other terms that the author or any third party may assert apply to any version of the manuscript.

Please note that Nature Research offers an immediate open access option only for papers that were first submitted after 1 January 2021.

If you have any questions about our publishing options, costs, Open Access requirements, or our legal forms, please contact ASJournals@springernature.com

If you have posted a preprint on any preprint server, please ensure that the preprint details are updated with a publication reference, including the DOI and a URL to the published version of the article on the journal website.

To assist our authors in disseminating their research to the broader community, our SharedIt initiative provides you with a unique shareable link that will allow anyone (with or without a subscription) to read the published article. Recipients of the link with a subscription will also be able to download and print the PDF.

As soon as your article is published, you will receive an automated email with your shareable link.

You can now use a single sign-on for all your accounts, view the status of all your manuscript submissions and reviews, access usage statistics for your published articles and download a record of your refereeing activity for the Nature journals.

An online order form for reprints of your paper is available at https://www.nature.com/reprints/author-reprints.html. Please let your coauthors and your institutions' public affairs office know that they are also welcome to order reprints by this method.

If you have not already done so, we invite you to upload the step-by-step protocols used in this

manuscript to the Protocols Exchange, part of our on-line web resource, natureprotocols.com. If you complete the upload by the time you receive your manuscript proofs, we can insert links in your article that lead directly to the protocol details. Your protocol will be made freely available upon publication of your paper. By participating in natureprotocols.com, you are enabling researchers to more readily reproduce or adapt the methodology you use. Natureprotocols.com is fully searchable, providing your protocols and paper with increased utility and visibility. Please submit your protocol to https://protocolexchange.researchsquare.com/. After entering your nature.com username and password you will need to enter your manuscript number (NG-BC57106R2). Further information can be found at https://www.nature.com/nprot/.

Sincerely,
Kyle

Kyle Vogan, PhD
Senior Editor
Nature Genetics
https://orcid.org/0000-0001-9565-9665
